# Case report: Reversible encephalopathy associated with liposomal amphotericin B in a patient with cryptococcal meningitis

**DOI:** 10.3389/fneur.2022.1019137

**Published:** 2022-11-09

**Authors:** Si Luo, Han Wen, Meihong Zhou, Chengsi Wu, Daojun Hong

**Affiliations:** Department of Neurology, The First Affiliated Hospital of Nanchang University, Nanchang, China

**Keywords:** liposomal amphotericin B, leukoencephalopathy, encephalopathy, cryptococcal meningitis, neurotoxicity

## Abstract

Liposomal amphotericin B (L-AMB) is an anti-fungus medicine that has fewer side effects than traditional amphotericin B (AMB). Neurotoxicity of L-AMB has rarely been observed, and only one case of leukoencephalopathy during intravenous L-AMB has been reported. Herein, we described a patient with cryptococcal meningitis presenting with late-onset reversible encephalopathy associated with liposomal amphotericin B.

## Introduction

Amphotericin B (AMB) is an effective medicine for fungal infections. Side effects of AMB such as nephrotoxicity, hypokalemia, and fever are common, but neurotoxicity is rarely observed (1–3). Neurotoxic symptoms of AMB may include confusion, incoherence, delirium, psychological behavior, tremors, convulsions, loss of hearing, flaccid quadriparesis, akinetic mutism, and rapidly progressive leukoencephalopathy, while those have only be observed in a few cases (4). Additionally, the factors associated with the complications and the evolutions of the symptoms are still unclear.

Liposomal amphotericin B (L-AMB) is a unique lipid formulation with fewer side effects than traditional AMB. Currently, only one case with intravenous L-AMB was reported to present with leukoencephalopathy mimicking acute disseminated encephalomyelitis (ADEM) (5). In this study, we described a patient with cryptococcal meningitis presenting with late-onset reversible abnormality of white matter in the central nervous system during withdrawal of L-AMB.

## Case report

**Figure 1:**
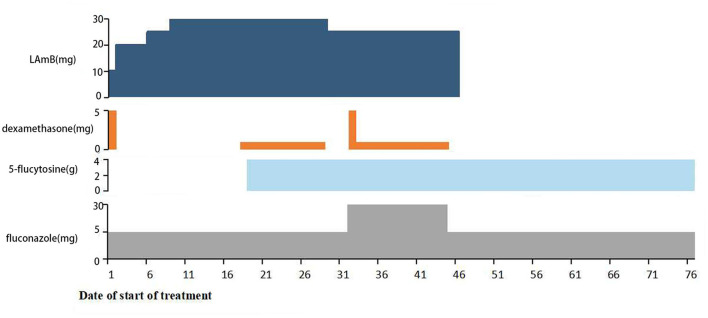
The medication of the patient during admission to our hospital. The patient first received induction therapy with L-AMB (10–30 mg/d) and fluconazole (400–800 mg/d), then followed by adding 5-flucytosine (25 mg/kg, 6-hourly) and intermittent use of Dexamethasone (1–5 mg/d).

A 39-year-old woman was referred to our department for headache and fever. On admission, brain magnetic resonance imaging (MRI) showed no abnormalities with white matter lesions or acute demyelinating lesions. The routine laboratory tests including a complete blood count, liver and renal function, and myocardial enzymes were almost normal. HIV and hepatitis C were negative in the patient. Cerebrospinal fluid (CSF) examinations revealed increased intracranial pressure of 250 mmH_2_O (normal 80–180 mmH_2_O), an increased protein level of 510 mg/L (normal 150–450 mg/L), an elevated white cell count of 70 /μl (normal 0–10/μl), and a low glucose level of 2.38 mmol/L (normal 2.8–4.4 mmol/L). The ink stain of CSF was positive; cryptococcal polysaccharide capsular antigen in the CSF was positive; and CSF culture was also positive to cryptococcus neoformans. Collectively, the final diagnosis was cryptococcal meningitis. The weight of the patient was 40 kilograms. Consequently, the patient was treated with L-AMB (10–30 mg/d, gradually increasing in the induction phase), 5-flucytosine (25 mg/kg, 6-hourly), and fluconazole (400–800 mg/d) according to the guideline in China (6). Dexamethasone (1–5 mg/d) was also used to alleviate inflammation-induced edema intermittently (Figure 1). After 34 days of anti-fungal therapy, the headache and fever of the patient rapidly improved. In addition, CSF culture was negative for Cryptococcus, and she was discharged home after a 48-day course of anti-fungal therapy. Then, she was switched to consolidation therapeutic schedule shifting from L-AMB to fluconazole (400 mg orally, BID) and 5-flucytosine (25 mg/kg 6-hourly) (Figure 1).

L-AMB was discontinued after the patient was discharged home. However, 30 days after discharge, or on the 78th day after the initial anti-fungal therapy with a cumulative dose of 1,115 mg L-AMB therapy course, she suddenly presented with recurrent seizure attacks without any prodromal symptoms. She suffered an abrupt onset of upper extremities twitching; the episodes last for about 1 min without loss of consciousness and then complete recovery. On the second day, she showed hallucinations, altered moods, disorganized speech, aggressive behaviors, and other psychiatric behaviors. Subsequently, she developed a decreased level of consciousness from confusion to lethargy and to coma. On the third day, the patient began to recover following dehydration, rehydration, and sedation treatment, she gradually became awake with return of consciousness. On the fourth day, the central nervous system symptoms completely disappeared, and the patient recovered completely. During this episode, physical examination revealed disorders of consciousness, but pupillary light reflex, Babinski sign, and signs of meningeal irritation were negative. After the episode, she was conscious, and the other clinical neurologic examinations were normal. In addition, after 1 and 3 months of discharged follow-up, the patient was symptom free without any recurrence.

The routine examinations including complete blood count, liver and renal function, lactate dehydrogenase, creatine kinase, myocardial enzymes, and thyroid function tests were negative. Lumbar puncture was performed to exclude the recurrence of cryptococcal meningitis. However, the cryptococcal polysaccharide capsular antigen, ink stain, and culture of CSF did not support cryptococcal meningitis. The examinations of blood and CSF related to autoimmune encephalitis, demyelination, and metabolic diseases were negative.

**Figure 2:**
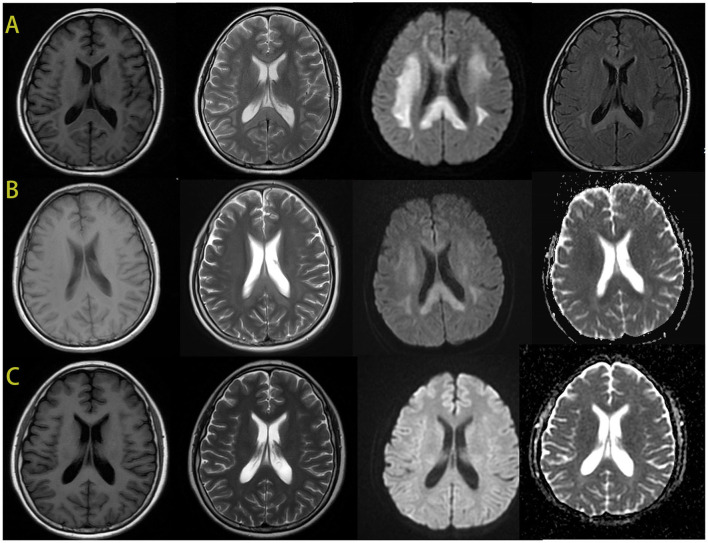
The brain MRI images of the patient. During the episode, the brain MRI showed hypointense signal in T1-weighted imaging and hyperintensity in T2-weighted imaging in the bilateral deep white matter and the corpus callosum. And there was an obviously bilaterally symmetric hyperintense DWI signal in the same region **(A)**. MRI after 4 days showed improvement of the abnormal signal in white matter, the hypointense ADC signal in the same region **(B)**. After 1 month, the images of her brain were almost normal **(C)**.

Cerebral MRI showed bilaterally symmetric hyperintensity on DWI signal and abnormal hyperintensity on T2 weight image in the deep white matter and corpus callosum (Figure 2). The abnormal signals gradually reduced and disappeared 4 days later. A month after the encephalopathy, her brain MRI was almost normal (Figure 2). The electroencephalogram (EEG) was normal during the episode.

## Discussion

We speculated the leukoencephalopathy of the patient might be related to the toxicity of L-AMB. First, there had not yet been any reports to present with the white matter lesions related to several other drugs that the patient had used, including fluconazole and 5-flucytosine. The fluconazole and 5-flucytosine had not been discontinued until the patient developed symptoms, and more importantly, they were still used between the episode and during recovery. However, some cases had reported that the leukoencephalopathy occurred with intravenous L-AMB (5). Therefore, we thought that it might possibly be related to the toxicity of L-AMB rather than fluconazole or 5-flucytosine. Additionally, the symptoms improved dramatically with non-specific treatment and the auto-antibodies relevant for demyelination and autoimmune encephalitis had not been found in the CSF and blood, thus the clinical and the imaging features did not support primary demyelinating disease or autoimmune encephalitis (7). Intriguingly, the leukoencephalopathy developed 30 days after the withdrawal of L-AMB. Bekersky et al. reported that the half-life of L-AMB elimination in tissues reached 1–4 weeks, which was longer than in plasma. In their study, rats received intravenous AMB for 91 days with a 30-day recovery. During recovery, the elimination of AMB from tissues was slower than its disappearance from plasma, and concentrations in liver and spleen greatly exceeded those in plasma (8). Therefore, it was reasonable to speculate that the half-life in brain tissue might be similar to liver and spleen tissues, and L-AMB might still be bio-available in the patient. Collectively, we thought that our patient might possibly have had one type of late-onset reversible leukoencephalopathy related to the toxicity of L-AMB. There were no similar reports of such cases, which could provide relevant reference for future clinical practice.

Cryptococcus meningitis could be associated with demyelinating leukoencephalopathy in a few cases. Wilcox et al. reported two cases of cryptococcus meningitis presenting with leukoencephalopathy prior to AMB therapy. They hypothesized that the lesions were induced by cryptococcal infection and parenchymal immune response (9). However, we thought that L-AMB, rather than cryptococcal infection itself, was suspected as a cause of leukoencephalopathy because CSF examination excluded cryptococcal meningitis recurrence in our patient. Additionally, mild encephalitis/encephalopathy with a reversible splenial lesion (MERS) or reversible splenial lesion syndrome (RESLES) should be differentiated from the late-onset reversible leukoencephalopathy. MERS was a clinical-radiological syndrome proposed by Tada in 2004 (10), and usually triggered by viral infections and bacterial infections in children (11, 12). Prodromal symptoms included fever, cold-like reactions, digestive tract disturbances, abdominal pain, and headache (13), and fever was considered the most frequent prodromal symptom since it had been detected in most case series (10, 14–16). The characteristic clinical and imaging findings of the patient showed some similarities with MERS. However, our patient was a 39-year-old adult, which did not indicate MERS, and the patient had no prodromal symptoms such as fever, headache, and suspected infections. RESLES was a clinical imaging syndrome that involved the splenium of the corpus callosum, and a small part of lesions could spread to adjacent white matter (17). RESLES had various causes, including infections, high-altitude cerebral edema, seizures and antiepileptic drug withdrawal, and metabolic disturbances. However, there were no predisposing factors related to RESLES in our patient, such as blood pressure fluctuations and special drugs (17). Furthermore, the imaging features in our patient were characterized by the involvement of the genu of the corpus callosum as well as diffused and symmetrical white matter lesions, which was very rare in RESLES cases. Therefore, our patient should not be considered as MERS or RESLES.

The neurotoxic potential of AMB included confusion, incoherence, delirium, psychiatric behaviors, tremors, convulsions, hearing loss, flaccid quadriparesis, akinetic mutism, and rapidly progressive leukoencephalopathy (4, 18). Most previous cases with central nervous system symptoms were either immune-compromised or had a history of radiotherapy (4, 19–22). However, our patient was a 39-year-old woman with cryptococcal meningitis and had no significant medical history. The nervous side effects of L-AMB had rarely been reported. Sato et al. described the first case of cryptococcal meningitis showing progressive leukoencephalopathy that was associated with L-AMB (5). Previous studies had reported that the leukoencephalopathy occurred while receiving intravenous L-AMB (1, 5), and the symptoms were usually severe after treatment with a high dose of L-AMB or AMB. However, our patient was initially treated with L-AMB combinations of fluconazole and flucytosine, followed by a month after discontinuation of intravenous L-AMB, and then the patient showed transiently reversible encephalopathic features. It was interesting that the encephalopathy symptoms were dramatically alleviated with dehydration, rehydration, and sedation treatment. Of note, AMB-associated leukoencephalopathy predominantly showed the white matter lesions closely associated with primary lesions (4, 23), while our patient presented with white matter lesions widespread cerebral white matter, and corpus callosum T2 hyperintensities on MRI.

Previous studies demonstrated that the pathogenic mechanism of AMB-associated leukoencephalopathy was correlated with the AMB binding to myelin, which led to an increase in membrane permeability and leakage of intracellular components, and eventually resulted in white matter lesion (4). The imaging characteristics of our patient showed hypointense signals on T1-weighted imaging and hyperintensity in T2-weighted imaging in the bilateral deep white matter and the corpus callosum and the hypointense ADC signal in the same region. Based on these characteristics, we also speculated that the pathogenesis might be due to L-AMB binding to myelin and leading to leakage of intracellular components, which then resulted in the cytotoxic edema and white matter lesions. However, the specific pathogenic mechanism needs to be confirmed by more cases.

Previous studies had reported that the leukoencephalopathy occurred while receiving intravenous L-AMB, and the symptoms were usually severe (1, 5). In contrast, our patient experienced acute encephalopathy during withdrawal of L-AMB, and the symptoms were mild, and the patient quickly showed dramatic improvement. This type of late-onset encephalopathy happening after withdrawal of L-AMB was very unusual, and had not been reported before. We speculated that the severity and onset of symptoms might be related to the dose and duration of L-AMB. More studies are warranted to confirm and better characterize these potentially important associations.

In summary, L-AMB had a significantly improved toxicity profile compared with AMB, while L-AMB-related leukoencephalopathy could be induced after withdrawal of L-AMB treatment. The late-onset reversible leukoencephalopathy might provide relevant reference for future clinical practice.

## Data availability statement

The raw data supporting the conclusions of this article will be made available by the authors, without undue reservation.

## Ethics statement

The studies involving human participants were reviewed and approved by the Ethics Committee of the First Affiliated Hospital of Nanchang University. Written informed consent to participate in this study was provided by the patient. Written informed consent was obtained from the patient for the publication of any potentially identifiable images or data included in this article.

## Author contributions

SL, HW, and CW were responsible for acquisition of data and drafting the manuscript. MZ was responsible for revising the manuscript. SL and DH were responsible for study concept or design, drafting, and revising the manuscript. All authors have read and approved the final manuscript.

## Funding

This work was supported by the National Natural Science Foundation of China (Grants 81460199 and 82160252), Natural Science Foundation of Jiangxi Province (20202BAB206029), and Double Thousand Talents Program of Jiangxi Province (jxsq2019101021).

## Conflict of interest

The authors declare that the research was conducted in the absence of any commercial or financial relationships that could be construed as a potential conflict of interest.

## Publisher's note

All claims expressed in this article are solely those of the authors and do not necessarily represent those of their affiliated organizations, or those of the publisher, the editors and the reviewers. Any product that may be evaluated in this article, or claim that may be made by its manufacturer, is not guaranteed or endorsed by the publisher.
